# Quick fix for care, productivity, hygiene and inequality: reframing the entrenched problem of antibiotic overuse

**DOI:** 10.1136/bmjgh-2019-001590

**Published:** 2019-08-15

**Authors:** Laurie Denyer Willis, Clare Chandler

**Affiliations:** Global Health and Development, London School of Hygiene & Tropical Medicine, London, UK

**Keywords:** antimicrobial resistance, care, productivity, hygiene, inequality

## Abstract

Antimicrobial resistance (AMR) is a major challenge of our time. A key global objective is to reduce antibiotic use (ABU), in order to reduce resistance caused by antimicrobial pressure. This is often set as a ‘behaviour change’ issue, locating intervention efforts in the knowledge and attitudes of individual prescribers and users of medicines. Such approaches have had limited impact and fall short of addressing wider drivers of antibiotic use. To address the magnitude of antibiotic overuse requires a wider lens to view our relationships with these medicines.

This article draws on ethnographic research from East Africa to answer the question of what roles antibiotics play beyond their immediate curative effects. We carried out interviews, participant observation and documentary analysis over a decade in northeast Tanzania and eastern and central Uganda. Our findings suggest that antibiotics have become a ‘quick fix’ in our modern societies. They are a *quick fix for care* in fractured health systems; a *quick fix for productivity* at local and global scales, for humans, animals and crops; a *quick fix for hygiene* in settings of minimised resources; and a *quick fix for inequality* in landscapes scarred by political and economic violence. Conceptualising antibiotic use as a ‘quick fix’ infrastructure shifts attention to the structural dimensions of AMR and antimicrobial use (AMU) and raises our line of sight into the longer term, generating more systemic solutions that have greater chance of achieving equitable impact.

**Summary box**

- Antimicrobial resistance (AMR) is a major challenge of our time. A key global objective is to reduce antibiotic use, in order to reduce resistance caused by antimicrobial pressure. Many interventions aimed to lower antibiotic use are based on models of behaviour change.
- Understanding antibiotic use in low-income and middle-income countries requires shifting attention to the structural dimensions of AMR and AMU that tend to be obscured when following an individual behaviour change approach.
- Antibiotics function as a ‘quick fix’. They are a quick fix for care in fractured health systems; a quick fix for productivity at local and global scales, for humans, animals and crops; a quick fix for hygiene in settings of minimised resources; and a quick fix for inequality in landscapes scarred by political and economic violence.
- Recognising many of our AMR solutions as quick fixes allows us to raise our line of sight into the longer term, generating more systemic solutions that have greater chance of achieving equitable impact.

## Introduction

Antimicrobial resistance (AMR) is a major challenge of our time. Governments around the globe are being encouraged to commit resources to tackle this issue. A key global objective is to reduce antibiotic use, in order to reduce resistance caused by antimicrobial pressure.^1 2^ Set out as a One Health problem, interventions are aimed at reducing antimicrobial use in human health, agriculture and in the environment.^1 3^ Low-income and middle-income countries (LMICs) have been identified as a specific target for AMR and antibiotic use policies due to a range of factors that locate them as particularly vulnerable to the effects of AMR,^4 5^ as well as the perception of them as posing a risk to other countries through the connectivity rendered so apparent in previous pandemic scares.^6 7^

How best to reduce antibiotic use remains a challenge. To date, attempts to reduce antibiotic prescribing and use have had mixed effects,^8–10^ and calls have been made for social research to understand why antibiotic use is so entrenched.^11^ There is a growing literature that explores the reasons for patients’ or doctors’ preferences for antibiotic use, such as knowledge, attitudes and incentives.^12 13^ This paper aims to bring into view the wider problems that these medicines have become solutions for, foregrounding the structural issues that contribute to widespread antimicrobial use that often go unaddressed in conceptions of antibiotic use that hinge on ‘good’ and ‘bad’ individual behaviours.^14 15^

Drawing on long-term anthropological study, our research explores how antibiotics have become interwoven with the ways societies and economies work and proposes that antibiotics have become *infrastructural*. By ‘infrastructure’ we draw on the work of Bowker and Star,^16^ who conceive of infrastructures as systems that ‘disappear almost by definition. The easier they are to use, the harder they are to see’ (p. 33). With antimicrobial resistance emerging as a major topic of global concern, the myriad ways that antimicrobials function as infrastructure are suddenly rendered visible, where previously they have been a part of the woodwork.

This is particularly palpable In LMICs, where antimicrobials function as a ‘quick fix’ infrastructure, put to work to correct the fractured infrastructures of care, water and sewage, hygiene and demands for ever increasing productivity. In this way, seeing antimicrobials as infrastructure can also ‘reveal forms of political rationality that underlie technological projects’^17^ (p. 328), demonstrating how neoliberal reforms, the legacies of structural adjustment programmes and the marginalisation of the poor and vulnerable have made antimicrobials an infrastructure that undergirds complex livelihoods in landscapes of scarcity, uncertainty and inequality.

**Figure 1:**
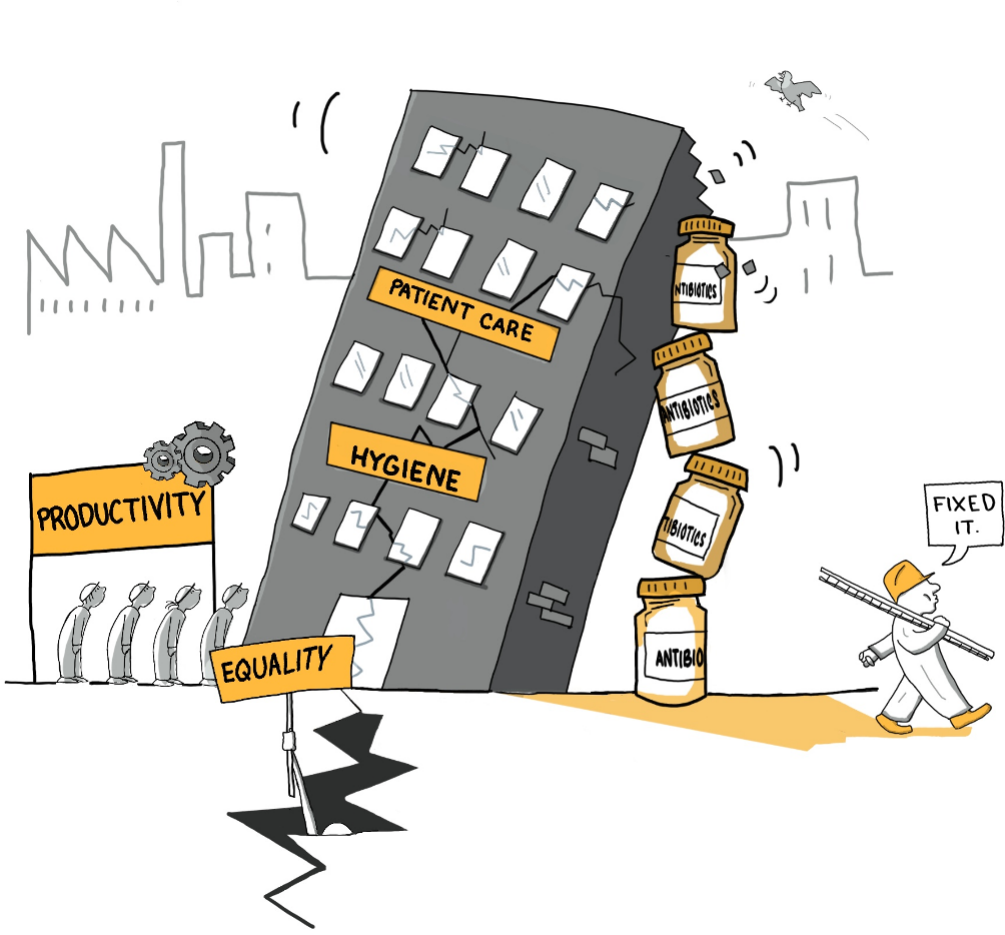
Antibiotics as a quick fix.

This piece, then, provides a means by which to shift attention towards these structural dimensions related to AMR and antimicrobial use (AMU) that tend to be obscured when following an individual behaviour approach. We do this by focusing on the work that antibiotics do for the wider systems in which people are making their lives, as depicted in figure 1. We provide ethnographic vignettes to illustrate our points, selected from our research in Uganda and Tanzania. We first present how antibiotics can be understood as a *quick fix for care* and, by extension, a *quick fix for productivity*. We then explore how antibiotics have become a *quick fix for hygiene* in some settings and more broadly a *quick fix for inequality* on local and global scales.

This research is based on long-term ethnographic research, including participant observation and key informant interviews, focusing on the ways that antimicrobial medicines are used in everyday life, and the contexts—political, social and economic—that underscore use. Analysis involved collaboration of the authors with key stakeholders and wider research team members.

## Quick fix for care and productivity

Antibiotic overuse is often described in terms of inappropriate ‘patient demand’ for antibiotics. The vignette in Box 1 can easily be read in these terms: patients demanding medicines to fit with their own needs. However, it can also be read as a struggle to provide care and to be productive, eased through use of antimicrobial medicines. Estimates of unnecessary antibiotic prescriptions suggest around 50% of prescriptions and purchases around the world are not needed.^18^ The explanation that this is due to demanding patients produces interventions that require patients to better understand the limits of what they can expect from healthcare providers, to stop pressuring them;^19^ and that require healthcare providers to employ defensive strategies as antibiotic gate-keepers.

Box 1 **Antibiotics as a quick fix for care and productivity**

After driving off-road for an uncomfortable 2 hours, the glimpse of the dispensary is a welcome sight. We have arranged our visit ahead of time, knowing that primary healthcare facilities are often closed, even in the morning hours. We are starting a research study about medicines and diagnosis for fever in this remote area of northeastern Tanzania. The nurse in charge is busy, and we wait outside together with a group of mothers and children assembled on benches and on the floor of the veranda. Eventually, the exhausted nurse emerges, and explains, ‘we are too busy; I have medicines this week, and the villagers know’. She says that patients often only come when medicines are there. Without medicines and patients they tend to close the dispensary and attend to their other small businesses, usually a *shamba* (back-yard farm), sometimes motorbike taxis. In the village, we hear similar accounts—the expectations from health workers is to provide medicines. If one medicine does not work, health workers provide another. This is what care is; medicines. A couple of years later, after the dispensary participated in a trial to reduce unnecessary antimalarial use through the provision of rapid diagnostic tests, we hear of the challenge of denying medicines. Health workers tell us that even if the test means that we cannot give an antimalarial, then we can give an antibiotic. This is borne out in subsequent quantitative analyses; a reduction in antimalarial use with rapid diagnostic testing leads to an increase in antibiotic use.^50 51^ Here, as in many places, it is hard to imagine care without medicines. And often this village lacks both.

Healthcare is a means to an end here. As in many places around the world, life requires work; a day’s lost labour has immediate consequences for the whole family.^52^ Here, medicines enable day wage labourers to make it through the day at the local plantation, enable the *shamba* to be tended and give the charcoal and water cyclists the strength to haul their loads through the sandy tracks. At this local level, in a context of razor-thin margins for survival, antibiotic medicines enter into the balance of a price to pay for productivity.

Research has shown, however, that such interventions are challenging to implement and can lead to unintended consequences. When healthcare providers were asked to limit antimalarial use through the deployment of rapid diagnostic tests in Uganda and Tanzania, for example, patients were rendered either ‘deserving’ or not of medicines and, by extension, of care itself.^20 21^ This outcome requires us to revisit assumptions about what patients are actually ‘demanding’ when they request or expect medicines. Is it possible that healthcare has been stripped down to the provision of medicines, such that to demand medicines is to demand care? In settings where access to physicians and nurses is severely constrained, what is care without medicines?

Multiple scholars have described a process of *pharmaceuticalisation* within biomedicine globally; a shift away from techniques and practices of prevention and clinical care to pharmaceutical intervention and treatment, such that primary public health and healthcare is defined in terms of access to pharmaceuticals.^22 23^ This is apparent in curricula for the increasing cadres of non-physician health workers in LMICs who are trained to enact simple algorithms of care that culminate in provision (or not) of particular medicines.^24^ Here we can see antimicrobial medicines, rather than clinical attentiveness, being written in to assessment and expectations of the healthcare process,^25^ extending to LMICs a trend previously documented in high income countries.^26^ Solidified as key healthcare deliverables for public sectors in LMICs through the ‘essential medicines’ movement in the 1970s, and consolidated in the subsequent neoliberal reforms that encouraged the opening up of the private sector to deliver health commodities from the 1980s onwards, antimicrobials can be understood to have become a technical *quick fix for care* in lieu of addressing what are known to be wider political-economic challenges that drive ill-health, fragmented disease prevention efforts and fractured health service delivery systems.^27^

The vignette in Box 1 also draws our attention to the local economic context in which antimicrobials are being consumed; here we see how these medicines enable people’s productivity—by keeping their bodies productive. We also often hear of the use of antibiotics to improve productivity of animals and fish as growth promoters and to increase yields through mass treatment and reduced risks of infection.^2^ The use of antimicrobials in crops is also becoming better understood for fungal pest control and use of antibiotics for bacterial infections, for example, of oranges in Thailand.^28^

Across all these bodies—human, animal, fish and plant—we can understand antimicrobials to be enabling a standardised unit of productivity. For humans, the potential loss to productivity of reduced antimicrobial efficacy was a key driver in the enormous economic losses projected in the O’Neill^29^ and World Bank^30^ reports on the risks of AMR. For animals, the use of antimicrobials enables standard sized animals, fish and standard-looking fruit to be generated and traded in global economic circuits. This clear link between antimicrobials and economies exposes the way that antimicrobials have become integrated with our systems—a *quick fix for productivity*—that enables the standardisation and predictability of labour that are required in global economic systems.

## Quick fix for hygiene and inequality

Individuals, carers and health workers in the Global South often find themselves using and prescribing antibiotics because of infections caught due to unsanitary conditions in healthcare settings and at home, as well as in anticipation of such infections.^31 32^ The reasons for such prophylactic antimicrobial prescribing are known to be complex^33 34^ but in part can be understood as an extension of infection prevention and control measures. This prophylactic use is necessary, many explain, because of inadequate sterilisation of patient rooms and medical tools in hospitals, lack of hospital beds and overcrowding and the unhygienic living and working conditions of patients themselves.

Our ongoing research in Uganda and Thailand underscores that antibiotic use is often a response to patients’ lack of access to clean water and sanitary toileting due to inadequate infrastructural provision in informal settlements and/or work under precarious and unhygienic conditions, as street vendors, household cleaners or labourers in industrial factories. Under these conditions, we observe that antibiotics function as a *quick fix for lack of hygiene*, acting as substitutes for the non-hygienic conditions that health workers and individuals work and live within.

Research to date has demonstrated how good hygiene practices in hospitals, clinics and everyday life can prevent infection,^35 36^ but the methods and/or interventions aimed at attaining those good sanitary conditions have often been scaled at the level of the individual, focused on changing individual hygiene behaviours. For example, many initiatives focus on hand washing and hygiene knowledge and awareness campaigns for health workers and the public.^15 37 38^ Achieving behavioural changes like hand hygiene, however, is not straightforward,^39 40^ often failing due to a lack of resources, fractured infrastructures and competing priorities.^41 42^

The saddling of responsibility for hygiene with individuals who have limited ability to change the environment in which ‘good hygiene behaviour’ is expected to operate leaves these individuals to find solutions that are more feasible and within their control, such as the use of antibiotics. As the vignette in Box 2 demonstrates, hygiene is rarely an individual issue. A lack of hygiene can also be understood as emerging from the inequitable distribution of what is required to be hygienic. In the Global South, when we think about inadequacies in hygiene systems, we must consider its relationship to structural violence, along with the marginalisation and criminalisation of the poor.^43^

Box 2 **Antibiotics as a Quick Fix for Hygiene and Inequality**

Grace brings us into her house to show us her plastic basket of medicines she keeps on hand at home. It is a small collection of Panadol (paracetamol), some amoxicillin tablets and a full box of metronidazole. She explains that the metronidazole is an absolute necessity here in this urban informal settlement in downtown Kampala, Uganda, where chronic diarrhoea—and the serious abdominal pain associated with it—is a condition that many adults and children live with every day. Walking through the settlement with her we pass the few existing fee-for-use latrines in the community, but Grace explains that most cannot afford them, and that regardless, many avoid them because they are so unclean. She explains that many people—including her—simply use home bucket systems or polythene bags for toileting, and then dispose of their waste either in the open drainage channels that run through the settlement or in community rubbish piles. These are the same drainage channels where people must wash their clothes and collect water for their livestock and gardens. Further exacerbating this situation, the settlement is built on and beside a wetland area, and these drainage channels often flash flood during the rainy season, with waters rising to knee-height and circulating inside people’s homes, their livestock pens and their small gardens. Here, antibiotics are put to use to make unavoidable diarrhoeal disease bearable, enabling people to carry on with their work and family commitments despite chronic diarrhoea and abdominal pain. There is little political will to tackle these issues, as the local press describes the informal settlement as a problematic ‘slum’, while the people who live there are cast as ‘illegal squatters’ and criminals. Here, antibiotics provide a quick fix for wider structural and political issues, manifested as lack of hygiene in contexts of poverty.

This entails a historical consideration of how colonial ideas about the ‘backwardness’ and ‘uncivilised’ *behaviours* of colonised peoples continues to shape global health policy and interventions today.^44 45^ For example, when clean water is not made available to certain populations,^46^ when public hospitals are underfunded and left to decline and make do without necessary products and materials,^47^ or when affordable housing is inaccessible, and people’s solutions to housing crises—like informal settlements—are deemed ‘illegal’ and ‘disordered’,^48^ access to sanitary infrastructure is *made* unequal. This is not about individual behaviour, but is often imagined as such.

As the vignette from Box 2 depicts, when antibiotics are deployed as a quick fix for hygiene inadequacies, they are being used as a technical fix in these constrained settings beset by inequality, where other options are made limited or unavailable. When hygiene is approached as a technical gap or a behaviour change issue, we risk actively obscuring inequality. This analysis suggests that antibiotic use as a *quick fix for hygiene* cannot easily be disentangled from the use of antibiotics as a *quick fix for inequality*.

The lure of antibiotics as a magic bullet to fix these systemic challenges is strong and made evident in the promise of recent trials that have indicated the potential for the mass distribution of antibiotics to preschool children to lower mortality rates in countries that are far from reaching the goal posts of the sustainable development goals (SDGs).^49^ Here, we can understand antibiotics as a *quick fix* for the entrenched inequalities that have made reaching the SDGs so tricky in the first place. By cutting through these barriers, the prophylactic use of broad-spectrum antibiotics depoliticises the unequal causes of morbidity and mortality, enabling political achievements at a far lower cost than would be required to change the conditions that necessitated the SDGs in the first place.

## Conclusion

This paper uses the concept of the *quick fix* as a way to recognise the limits of our vision on solutions to AMR. By attending to antibiotics as a *quick fix*, we show how these substances can usefully be understood as a kind of gap-filling infrastructure that performs certain kinds of work in our societies—often replacing people, attentiveness, political change, recuperation and technical infrastructures. Antibiotics are deployed to paper over long-term structural issues that undermine care provision, drive increased productivity and correct for hygiene issues caused by entrenched inequality. We attend to antibiotics here as having themselves become a kind of infrastructure that enables modern life.

This approach shifts the conversation away from the more commonplace binary of ‘appropriate/inappropriate’ antibiotic use. Instead we can ask how else we might take seriously and care for people defined beyond bodies and pathogens? How else might we imagine our systems of healthcare, infrastructure and political-economies, such that we can take seriously and care for our populations beyond units of productivity? The importance of this type of analysis lies in its ability to render visible the reasons that antimicrobial use is so entrenched in our societies.

As we develop new modes of thinking about antibiotic use, it will inevitably be tempting to try to replace one quick-fix for another, or to dismiss critical analyses as possibly impractical or too costly. This need not be the case. Recognising many of our AMR solutions as *quick fixes* allows us to raise our line of sight into the longer term, generating more systemic solutions that have greater chance of achieving equitable impact. This will necessarily involve rethinking assumptions about so-called individual behaviours and allow us to consider how structural solutions, which provide the scaffolding for more equitable health and care, can be developed, funded and implemented. *How* we understand the issue of antimicrobial use matters if we want to successfully deal with the consequences of AMR for modern medicine.

## References

[1] World Health Organisation Global action plan on antimicrobial resistance. Geneva: Available online at, 2015.

[2] OIE The OIE strategy on antimicrobial resistance and the Prudent use of antimicrobials. World Organisation for Animal Health, 2016 Online at.

[3] Food and Agiculture Organisation of the United Nations The Fao action plan on antimicrobial resistance 2016-2020. Rome. Available online at 2016 http://www.fao.org/3/a-i5996e.pdf

[4] Okeke IN, Laxminarayan R, Bhutta ZA, et al Antimicrobial resistance in developing countries. Part I: recent trends and current status. Lancet Infect Dis 2005;5:481–93. 10.1016/S1473-3099(05)70189-4 16048717

[5] Alvarez-Uria G, Gandra S, Laxminarayan R Poverty and prevalence of antimicrobial resistance in invasive isolates. International Journal of Infectious Diseases 2016;52:59–61. 10.1016/j.ijid.2016.09.026 27717858

[6] Holmes AH, Moore LSP, Sundsfjord A, et al Understanding the mechanisms and drivers of antimicrobial resistance. The Lancet 2016;387:176–87. 10.1016/S0140-6736(15)00473-0 26603922

[7] Talkington K. Superbugs Don’t Respect Borders Combating the growing threat of antibiotic resistance must remain a top global priority. 2017 Available: https://www.pewtrusts.org/en/research-and-analysis/articles/2017/10/10/superbugs-dont-respect-borders

[8] Price L, Gozdzielewska L, Young M, et al Effectiveness of interventions to improve the public’s antimicrobial resistance awareness and behaviours associated with prudent use of antimicrobials: a systematic review. J Antimicrob Chemother 2018;73:1464–78. 10.1093/jac/dky076 29554263

[9] Arnold SR, Straus SE, Cochrane Effective Practice and Organisation of Care Group Interventions to improve antibiotic prescribing practices in ambulatory care. Cochrane Database of Systematic Reviews 2005;20 10.1002/14651858.CD003539.pub2 PMC7003679 16235325

[10] Davey P, Marwick CA, Scott CL, et al Interventions to improve antibiotic prescribing practices for hospital inpatients. Cochrane Database of Systematic Reviews 2017;39 10.1002/14651858.CD003543.pub4 PMC6464541 28178770

[11] Macintyre S Anti-Microbial resistance: setting the social science agenda. Report of an ESRC Working Group, 2014 Available online at.

[12] MaCIRC P Knowing antimicrobial resistance in practice: a multi-country study with human and animal healthcare professionals. Global Health Action 2019. 10.1080/16549716.2019.1599560 PMC6703149 31294679

[13] Chandler CIR, Hutchinson E, Hutchison C Addressing antimicrobial resistance through social theory: an Anthropologically oriented report: London school of hygiene and tropical medicine, 2016 and.

[14] Collignon P, Beggs JJ, Walsh TR, et al Anthropological and socioeconomic factors contributing to global antimicrobial resistance: a univariate and multivariable analysis. The Lancet Planetary Health 2018;2:e398–405. 10.1016/S2542-5196(18)30186-4 30177008

[15] Vindigni SM, Riley PL, Jhung M Systematic review: handwashing behaviour in low- to middle-income countries: outcome measures and behaviour maintenance. Trop Med Int Health 2011;16:466–77. 10.1111/j.1365-3156.2010.02720.x 21226794

[16] Bowker GC, Star SL Sorting things out. classification and its consequences. Cambridge Massachusetts: MIT Press, 2000.

[17] Larkin B The politics and Poetics of infrastructure. Annu Rev Anthropol 2013;42:327–43. 10.1146/annurev-anthro-092412-155522

[18] World Health Organisation Medicines use in primary care in developing and transitional countries. fact book summarizing results from studies reported between 1990 and 2006. Geneva 2009.

[19] Glover R, Dangoor M, Mays N Educating patients or blaming them? public education campaigns on antibiotic resistance. BMJ (clinical research ED), 2019 Available: https://blogs.bmj.com/bmj/2019/02/01/educating-patients-or-blaming-them-public-education-campaigns-on-antibiotic-resistance/10.1136/bmj.l1218 30890532

[20] Chandler CIR, Webb EL, Maiteki-Sebuguzi C, et al The impact of an intervention to introduce malaria rapid diagnostic tests on fever case management in a high transmission setting in Uganda: a mixed-methods cluster-randomized trial (prime). PLoS One 2017;12:e0170998 10.1371/journal.pone.0170998 28288172 PMC5347994

[21] Hutchinson E, Reyburn H, Hamlyn E, et al Bringing the state into the clinic? incorporating the rapid diagnostic test for malaria into routine practice in Tanzanian primary healthcare facilities. Global public health 2015:1–15. 10.1080/17441692.2015.1091025 PMC5526135 26457440

[22] Bell SE, Figert AE Moving Sideways and Forging Ahead. Reimagining "-Izations" in the Twenty-First Century : Bell SE, Figert AE, Reimagining (Bio)Medicalization, Pharmaceuticals and Genetics. New York: Routledge, 2015: 19–40.

[23] Biehl J Pharmaceutical Governance : Petryna A, Lakoff A, Kleinman A, Global pharmaceuticals: ethics, markets, practices. Durham, NC: Duke University Press, 2006.

[24] Gouws E, Bryce J, Habicht JP, et al Improving antimicrobial use among health workers in first-level facilities: results from the multi-country evaluation of the integrated management of childhood illness strategy. Bulletin of the World Health Organization 2004;82:509–15. 15508195 PMC2622903

[25] Opening up ‘fever’, closing down medicines: algorithms as blueprints for global health in an era of antimicrobial resistance Medicine Anthropology Theory Forthcoming

[26] Macfarlane JT, Worboys M The changing management of acute bronchitis in Britain, 1940–1970: the impact of antibiotics. Med Hist 2008;52:47–72. 10.1017/S0025727300000156 18180811 PMC2175052

[27] Petryna A, Lakoff A, Kleinman A Global Pharmaceuticals: Ethics, Markets, Practices. London: Duke, 2006.

[28] Urapeepathanapong T, Chawraingern S, Hutchison C Antibiotic Angels: Seeing Green in Thailand’s Orange Orchards. AMIS Hub, 2018.

[29] O'Neill J Tackling drug-resistant infections globally: final report and recommendations. London: The Review on Antimicrobial Resistance, 2016.

[30] World Bank Group Drug-Resistant infections. A threat to our economic future. Washington, DC. Available online at 2017 www.worldbank.org

[31] Pearson MDA, Glogowski R, Ibezim S, et al And resistance: views from LMIC prescribing and dispensing professionals. Report to World Health Organisation AMR Secretariat 2018.

[32] AaHH R Improving antibiotic use in low-income countries: an overview of evidence on determinants. Social Science and Medicine 2003;57:733–44. 12821020 10.1016/s0277-9536(02)00422-7

[33] Chandler CIR, Jones C, Boniface G, et al Guidelines and mindlines: why do clinical staff over-diagnose malaria in Tanzania? A qualitative study. Malar J 2008;7:53 10.1186/1475-2875-7-53 18384669 PMC2323020

[34] Kamat VR “I thought it was only ordinary fever!” cultural knowledge and the micropolitics of therapy seeking for childhood febrile illness in Tanzania. Soc Sci Med 2006;62:2945–59. 10.1016/j.socscimed.2005.11.042 16403595

[35] Luby SP, Agboatwalla M, Feikin DR, et al Effect of handwashing on child health: a randomised controlled trial. The Lancet 2005;366:225–33. 10.1016/S0140-6736(05)66912-7 16023513

[36] Waddington H, Snilstveit B Effectiveness and sustainability of water, sanitation, and hygiene interventions in combating diarrhoea. Journal of Development Effectiveness 2009;1:295–335. 10.1080/19439340903141175

[37] Allegranzi B, Gayet-Ageron A, Damani N, et al Global implementation of who's multimodal strategy for improvement of hand hygiene: a quasi-experimental study. Lancet Infect Dis 2013;13:843–51. 10.1016/S1473-3099(13)70163-4 23972825

[38] Biran A, Schmidt W-P, Varadharajan KS, et al Effect of a behaviour-change intervention on handwashing with soap in India (SuperAmma): a cluster-randomised trial. The Lancet Global Health 2014;2:e145–54. 10.1016/S2214-109X(13)70160-8 25102847

[39] Langford R, Panter-Brick C A health equity critique of social marketing: where interventions have impact but insufficient reach. Soc Sci Med 2013;83:133–41. 10.1016/j.socscimed.2013.01.036 23452864

[40] Willmott M, Nicholson A, Busse H, et al Effectiveness of hand hygiene interventions in reducing illness absence among children in educational settings: a systematic review and meta-analysis. Arch Dis Child 2016;101:42–50. 10.1136/archdischild-2015-308875 26471110 PMC4717429

[41] Horton S, Barker JC “Stains” on their self-discipline: Public health, hygiene, and the disciplining of undocumented immigrant parents in the nation's internal borderlands. Am Ethnol 2009;36:784–98. 10.1111/j.1548-1425.2009.01210.x 20161433 PMC2787473

[42] Joshi D, Fawcett B, Mannan F, Health MF Health, hygiene and appropriate sanitation: experiences and perceptions of the urban poor. Environ Urban 2011;23:91–111. 10.1177/0956247811398602

[43] Farmer P Pathologies of power: health, human rights, and the new war on the poor. Berkeley University of California Press, 2004.

[44] Benton A, Sangaramoorthy T, Kalofonos I Temporality and positive living in the age of HIV/AIDS: a Multisited ethnography. Curr Anthropol 2017;58:454–76. 10.1086/692825 29075043 PMC5653251

[45] James E Democratic Insecurities: violence, trauma, and intervention in Haiti. Berkeley University of California Press, 2010.

[46] Anand N Pressure: the PoliTechnics of water supply in Mumbai. Cultural Anthropology 2011;26:542–64. 10.1111/j.1548-1360.2011.01111.x 22171410

[47] Pfeiffer J, Chapman R An anthropology of aid in Africa. The Lancet 2015;385:2144–5. 10.1016/S0140-6736(15)61013-3 26068257

[48] Roy A Slumdog cities: rethinking Subaltern Urbanism. Int J Urban Reg Res 2011;35:223–38. 10.1111/j.1468-2427.2011.01051.x 21542201

[49] Keenan JD, Bailey RL, West SK, et al Azithromycin to reduce childhood mortality in sub-Saharan Africa. N Engl J Med 2018;378:1583–92. 10.1056/NEJMoa1715474 29694816 PMC5849140

[50] Cundill B, Mbakilwa H, Chandler CIR, et al Prescriber and patient-oriented behavioural interventions to improve use of malaria rapid diagnostic tests in Tanzania: facility-based cluster randomised trial. BMC Med 2015;13:118 10.1186/s12916-015-0346-z 25980737 PMC4445498

[51] Hopkins H, Bruxvoort K, Cairns M, et al The impact of introducing malaria rapid diagnostic tests on antibiotic prescribing: a nine-site analysis in public and private health care settings. BMJ 2017.

[52] Kamat VR Silent violence. global health, malaria, and child survival in Tanzania. Tucson: University of Arizona Press, 2013.

